# Supplementary material for: Modeling treatment of osteoarthritis with standard therapy and senolytic drugs
Source: PLoS One. 2025 Sep 22;20(9):e0332763. doi: 10.1371/journal.pone.0332763 (PMC12453251; doi:10.1371/journal.pone.0332763)
Supplement: Attachment — Submitted filename: Response to Reviewers_OA.pdf [file pone.0332763.s001.pdf]

Response to Reviewer 1

This review highlights the manuscript's ambitious attempt to model a complex disease but identifies several critical issues that would need to be addressed before publication. The primary concerns center on model validation, parameter uncertainty, and the disparity between the mathematical and clinical applicability of the results.

The authors have undertaken a challenging modeling problem, but the work would benefit significantly from more rigorous validation approaches and better integration with clinical outcomes. The very low efficacy values (0.15-0.22%) compared to substantial clinical improvements (22-33% reduction in WOMAC scores) suggest that the model may not be capturing the most clinically relevant aspects of OA progression and treatment response.

Summary

This manuscript presents a mathematical model of osteoarthritis (OA) progression that incorporates cellular senescence and treatment effects of undenatured collagen type II (UC-II) and the senolytic drug fisetin. The authors develop separate models for males and females, validate them against clinical trial data, and conduct in silico trials to assess treatment efficacy and synergy.

Novel Integration: The incorporation of cellular senescence mechanisms into OA modeling is timely and biologically relevant, addressing a significant gap in current therapeutic approaches.

Sex-Specific Modeling: The differentiation between male and female models, particularly accounting for postmenopausal effects on collagen degradation and MMP-13 levels, is a valuable contribution.

Clinical Validation: The authors attempt to validate their model against four published clinical trials (T1-T4), demonstrating a correlation between computed E-efficacy and reported improvements in WOMAC scores. Extensive parameter estimation based on literature values and steady-state assumptions demonstrates thorough model development.

Major Comments

Comment 1. Model Assumptions and Simplifications

Critical Issue: The fundamental assumption that OA progression is solely measured by calcified cartilage density (Cc) is oversimplified and potentially problematic. OA is a complex, multifactorial disease involving:
Cartilage degradation beyond calcification, Synovial inflammation, Subchondral bone changes, and Pain mechanisms independent of structural damage. However, authors acknowledge this limitation but don't adequately address how this affects model validity.

Response

We thank the Reviewer for this comment. In this first mathematically developed OA model we aimed for a simple model that captured some significant features of the disease and its treatment. We therefore considered aging patients, who, in most cases, the OA is not inflammatory; we also made simple assumptions about the effect of subchondral bone changes and ignored pain mechanisms independent of structure damage. Detailed explanations are given in the Conclusion section, under model limitation #1.

Comment 2

The model contains numerous parameters (> 25) estimated from disparate sources, steady-state assumptions, and author assumptions; however, no sensitivity analysis is provided. For example, the choice of $\lambda_{Ch,Cc} = 10^4\ d^{-1}$ appears arbitrary without justification.

Response

We thank the reviewer for this comment. We performed sensitivity analysis (see Fig. 9). But as we mention in Conclusion, model limitation #2, the accuracy of some parameters is still questionable in the absence of clinical experiments.

Comment 3

The correlation between E-efficacy and WOMAC scores shows very low efficacy values (0.15-0.22%) for treatments that demonstrated improvements of 22-33% in WOMAC scores. This raises questions about the model's predictive validity and whether it captures meaningful therapeutic effects.

Furthermore, the relationship between senescent cell elimination and improvement in OA progression is assumed rather than established. Clinical trial data for fisetin show minimal effects, yet the model predicts synergy. The correlation between model predictions and clinical outcomes is weak.

Response

We thank the Reviewer for this comment.
Table 2 on the positive correlation between WOMAC score and UC-II treatment is NOT meant to suggest model validation. We added a few sentences in Pages 8–9 (in RED) to clarify that Table 2 demonstrates improvement in efficacy by UC-II treatment is positively correlated to reduced WOMAC score.

Eq. (3.19) defines the concept of synergy in a combination of UC-II and fisetin. Although the outcome of fisetin treatment is weak, it synergistically contributes in a combination with UC-II.

Minor Issues

Comment. The authors claim model validation based on four trials, but the correlation is weak.

Response. See our response to Comment 3 of Major Comments above. We also replaced "clinical trials" by "clinical studies" all through the paper.

Comment. Treatment protocols in Tables 4–6 don't match standard clinical practice, and the 6-month on/off UC-II protocol lacks clinical justification.

Response. In the clinical studies we used protocols based on available studies, as we now stated (in RED) at the beginning of the section "Clinical studies *in-silico*."

Comment. No confidence intervals or uncertainty quantification provided.
Comment. No statistical comparison between treatment groups

Response [we combine the later two comments] Since the results of the paper are not experimentally validated, we did not proceed to analyze confidence intervals and statistical comparison between treatment groups.

Comment. Key parameters like drug effectiveness coefficients lack experimental validation

Response. We agree with this comment, and addressed it the Conclusion section, model limitations #2 and #3.

Response to Reviewer 2

I have extensively read through the manuscript titled, "Modeling treatment of osteoarthritis with standard therapy and senolytic drugs", and I am highly impressed with the presentation of the work. The manuscript presents a comprehensive mathematical model of osteoarthritis (OA) incorporating biological mechanisms such as chondrocyte senescence, hypertrophy, calcified cartilage formation, and the roles of collagen type II and MMP13. It evaluates treatment effects using UC-II and senolytic drugs (fisetin), both independently and in combination, providing a novel in-silico framework validated against clinical trial data. The work is well-structured, technically rigorous, and contributes valuable computational insight into OA treatment planning.
However, here are some few suggested issues related to the work to improve it:

Comment 1

The study uses calcified cartilage (Cc) as a proxy for OA severity and compares it to WOMAC pain scores. The authors should explicitly discuss the biological or clinical rationale for using Cc density as a surrogate for clinical pain outcomes, even if only qualitatively.

Response

As stated in the Introduction, Grades 4,5,6 of OA were characterized by increasing the percentage of calcified cartilage. In the Conclusion, model limitation #1, we explained that OA is a complex disease, and that there are different ways to characterize its severity; for simplicity we chose the level of calcified cartilage.

Comment 2

While the mixed-type boundary condition is mathematically acceptable, more biological interpretation or justification would strengthen the modeling assumptions, especially for protein/drug diffusion.

Response

We thank the Reviewer for this comment.
We added interpretation of the boundary condition following Eqs. (2.14), (2.15), in BLUE. Since proteins are smaller than cells by 9 orders of magnitude, they presumably can easily leak out of the cartilage, whereas cells cannot.

Comment 3

Consider including a sensitivity analysis to identify which parameters have the greatest influence on model outcomes, particularly on efficacy metrics. Even a local sensitivity analysis would improve robustness.

Response

We thank the Reviewer for this comment.
We added a new Section 5 on "Parameter sensitivity analysis" (in RED).

Comment 4

In some places, there are unclear or potentially redundant terms in equations (e.g., repeated $\partial Ch/\partial t$ in Eq. 2.3). Carefully review all equations for typographical consistency.

Response

Done

Comment 5

Ensure that all variables shown in the schematic diagram (Figure 1) are labeled and defined in the caption or immediately adjacent text.

Response

Done.

Comment 6

Define all symbols the first time they appear (e.g., KL2 in Eq. 2.1), even if they are listed in Table 1. This will improve readability for interdisciplinary readers.

Response

We thank the Reviewer for this comment.
We made these additions; see Page 5, in BLUE.

Response to Reviewer 3

The article its good and the results it's correct so we can accept this article with minor correction Minor correction. The Authors must add recent references
10.3934/MATH.2023821
10.3390/sym15020286
10.1155/2022/2138165
10.1155/2022/5468696

Response

We thank the Reviewer for the comments. However, we note the following regarding the Reviewer's suggested references:
The first paper is about fractional evolution equations, is purely mathematical, the next two references are on modeling Covid-19 by fractional order equations, and the last one is an epidemical model with fractional order equations. All these references are not relevant to our manuscript, so we do not include them in our paper.
